# Singing in Churches

**Published:** 1870-04

**Authors:** 


### SINGING IN CHURCHES.

Not a few of those who read this article will confess that "many a time and oft," if the church choir had but one nose and that nose could be reached, a tremendous tweak of it could be given with a sweet satisfaction. You go to church of a Sunday morning; the minister gives out an old familiar hymn, a hymn which from time immemorial has been associated with a particular tune, in fact you had scarcely ever heard it sung to any other, and you feel as if you were ready to join in with most emphatic unction; and further, that you wanted to go ahead, without a moment's delay; in fact, you may have already made a start on your own responsibility, but coming to yourself, you find that the choir is not ready to begin; they are turning over their leaves, and whispering, and nodding away at a great rate, often smirking and grinning like a parcel of monkeys; after a while they get themselves adjusted, pass the key-note along the line, and go off in a tangent; the notes are strange, the chords grate terribly on the unaccustomed ear, and a feeling of blank and helpless disappointment comes over you; all the music in your soul has gone out at your finger-ends, your enthusiasm has vanished, and your anticipated enjoyment in singing the dear familiar strains is sacrificed and lost.

At other times, at the end of a feeling prayer or stirring address, when some familiar words are given out to be sung, words in unison with the occasion, and when there ought not to be a single moment's interval, so long a time is spent in getting ready, or some unfamiliar piece of music is attempted, that again all your enjoyment and enthusiasm is scattered to the winds, and if you feel at all, it is a very decided angry disappointment.

Song is the natural expression of worship and devotion ; a thousand times have we seen it, and elt it to be a cruelty and a despotism to have restrained a whole assembly, and forced them to listen to some unknown tune, when their real feeling, at the end of the discourse, prompted them to "break forth into singing" some old familiar strain.

Sometimes, after sitting in a kind of sullen silence until the close of the hymn, and the old Doxology of—

"Praise God from whom all blessings flow,"

is called for by the clergyman, there will be such a deep, hearty, and gushing joining in from every voice, it almost seems as if the roof would be raised from the building, as if the people could not keep their feelings pent up any longer, and as if there was a felt relief in the very loudness and depth of their voices. There are two other

IMPUDENT AND IMPERTINENT OUTRAGES

in the singing of religious worship. Every man who makes a hymn-book, and every man who publishes a collection of tunes, takes it upon himself to tinker with the words, and tinker with the notes, to suit his own stupidity and vanity, with the result, that often, when you are singing in a strange assembly, and fondly imagining that it is the same old hymn and tune which you had always sung at home, you are all at once brought to a sudden stand-still, by some strange word or strange note, which instantly spoils all your comfort and composure.

These things are particularly noticeable in the Presbyterian churches of New York city. It almost seems as if each congregation had its own hymns and its own tunes. Consequently, when one goes to any other than his own church, he feels like a fish out of water, and sits "mum" the whole time of service. This is a crying evil, and ought to be rectified. Every denomination should have the same hymn-book and the same tunes, and thus enable each worshipper to feel at home in the great congregation, whether in Maine or Florida, in California or Cape Cod.

### TOO MANY HYMNS.

There are entirely too many hymns and tunes in all our churches. It does the soul a thousand times more good to sing a familiar hymn to one particular tune; it helps the spirit of devotion; it helps spiritual enjoyment; and by the absence of all effort, the mind and heart have more leisure to contemplate the meaning of the words sung, and to be in unison with the sentiments expressed. In fact, it is only devotion when the mind is deeply impressed with the meaning of the words, but if it is diverted by an unusual word, or by an unusual note, it cannot be a worshipful singing; it cannot be the song of the heart, but of the head or intellect only.

There would be an important advantage in having a few hymns, and each hymn to have its own tune, and also have the tune named by the first few words of the first line, or by its sentiment as in —

"Ye banks and braes,"
"Oft in the stilly night,"
"Auld Lang Syne,"
"Coronation,"
"Sweet Home,"
"I want to be an angel,"
"Watchman, tell us of the night,"

"From Greenland's icy mountains."

In all these cases, the words, the tune, and the sentiment, rise spontaneously in the memory, with the most pleasurable associations.

But there is such a frequent change in the names of tunes, in the wording of hymns, and in the notes of the tunes, that out of our own individual church, we feel afraid to join in the music and the song, and thus lose a large share of the religious comfort which belongs to public worship.

Lowell Mason, the father of American Church Music, merits the thanks of all Christian people, for what he has done to purify, and elevate, and popularize it, as well as for the many sweet compositions which he has contributed, and with which he has greatly enriched our stores of song. He has made a move in the right direction by preparing

"THE AMERICAN TUNE BOOK,"

all of them old tunes, selected by 500 teachers and choir leaders, published by O. Ditson & Co., of New York. This admirable tune book merits three severe criticisms: —

The names of many tunes are changed.

The words are often changed.

The music is frequently altered.

What would Mr. Mason think if any man were to take the liberty of altering a single note in any one of his sweet compositions.

Suppose "Coronation" were called for in a public assembly, and on turning to the piece called by that name, you were to find that it was the same old tune that the muley cow died of.

Imagine that the

"SHINING SHORE"

was announced from the pulpit, and three thousand people were to join in with their minister, and with the unction peculiar to Mr. Beecher's church, seeming almost to raise the roof from the building, and after all had got under full headway singing, —

> Should coming days be dark and cold,
> We need not cease our singing,
> That perfect rest naught can molest,
> Where golden harps are ringing, —

the choir should sing instead, —

> Should coming days be dark and cold,
> We will not yield to sorrow,
> For hope will sing with courage bold,
> There is glory on the morrow, —

does not the reader think that the man who had enough of "courage bold," to make such a tremendous improvement on the original, ought to be elected commander-in-chief of all the geese in the galaxy of the skies, provided, however, there is such a galaxy, and a goose was fool enough to be straying around there.

As to the hymn-book to be used, the best selection ever made in accordance with our views, as already expressed, has been lately published by Hurd and Houghton, New York and Boston, entitled,

HYMNS FOR ALL CHRISTIANS, chosen from some twenty thousand metrical compositions by the Rev. Dr. Deems, and Phœbe Cary; a veritable book of psalms and hymns and spiritual songs; "lyrics" being used instead of psalms, as possessing a wider range of meaning. There are one hundred of each; a dollar book with a flexible cover, with paper, printing, size of type, etc., all unexceptionable. Now if each hymn had its own tune on the opposite page, we would vote that it should be exclusively used in the public worship of the Sabbath day, in every house of worship in Christendom.

There is one feature in it which will gratify many; the author of each hymn is given as far as known, and at the first mention of his name a very few items of personal history are given, or some incident connected with the hymn which adds to its impressiveness.

> "A charge to keep I have,
> A God to glorify,"

is the first hymn, ["by Charles Wesley, born 1708, died 1788."] The second hymn closes each verse with —

> "I will remember thee."

["By James Montgomery, England, born 1771, died 1854. A paraphrase of Luke xxii. 19."]

> "Alas, and did my Saviour bleed,"

is the third hymn, ["by Isaac Watts, D. D., a non-conformist minister, born 1674, died 1726, written in 1709."]

> "Guide me, O thou great Jehovah,"

["By Rev. Wm. Williams, of Wales, born 1717, died 1791."]

> "Jesus, these eyes have never seen,"

["By Ray Palmer, D. D., born in Rhode Island, 1808. Paraphrases 1 Peter i. 8."]

> "While Thee I seek, protecting Power,"

["By Mrs. H. M. Williams. We know nothing of the author of this marvellously fine hymn except her name, and that she was born in 1762, and died 1827. Can any one change a solitary word in the fourth stanza without marring it?"]

"Abide with me; fast falls the eventide."

["By Rev. Henry Francis Lite, born at Kelso in 1793, died in 1847 at Nice. This was his last hymn, written shortly before his death.']

"The Rock of my salvation,"

is the closing line of every stanza of a hymn, ["by Francis S. Key, born in Maryland, 1799, died in Washington in 1843. He is known as the author of the "Star-spangled Banner."]

"In evil long I took delight,"

["By Rev. John Newton, born in 1725, died in 1807. He was the friend and pastor of William Cowper. In this poem he records the history of his conversion."]

"O for a closer walk with God,"

["By William Cowper of England, born 1731, died in 1800. He was much of his life under the cloud of insanity; he wrote this hymn expressive of his spiritual darkness, in one of his lucid intervals."]

"Rock of Ages, cleft for me,"

["By Rev. Augustus M. Toplady, an English clergyman, born 1741, died 1778. A favorite with every Christian who has ever heard it. Prince Albert used it in his dying hour. The author of 'Rock of Ages,' and the author of

'Jesus, Lover of my soul,'

were fierce polemics while they lived, carrying their warfare into personalities. They had zeal in prose, but charity in poetry. Who doubts but that each has found a 'Refuge for his soul,' in 'The Rock of Ages.'"]

"Come, ye disconsolate,"

["By Thomas Moore, born in Ireland 1780, died in 1852."]

Doubtless some of our readers would be glad to read for an hour longer such annotations as these; they are not only instructive, but add interest to the hymn.

As confirmatory of one of our positions that there is devotional advantage in singing certain words to certain tunes, the following note precedes —

"Before Jehovah's awful throne,"

["By Dr. Watts. Let this hymn never be sung to any other tune than 'Old Hundred.'"]

We must add a few more.

"Nearer, my God, to Thee,"

["By Sarah Francis Adams, who died in 1848."]

"Just as I am, without one plea,"

["By Miss Charlotte Elliott of England."]

How many Christian souls now in heaven, and countless thousands on earth, have sweetly sung —

"Come, thou Fount of every blessing,"

["By Rev. Robert Robinson, an eccentric English Independent, once a follower of Whitfield. This favorite hymn was written in early life. He afterwards became irreligious. One day, while travelling in a stage-coach, a lady, not knowing who he was, called his attention to this hymn in a book she was reading. He endeavored to change the conversation. When she reverted to the subject, he burst into tears, and said, 'Madam! I am the unhappy man who wrote that hymn, and I would give a thousand worlds to enjoy the feelings I then had.' "]

"I love thy kingdom, Lord,"

["By Rev. Timothy Dwight, D. D., born in Massachusetts, 1752, died 1817. Paraphrase of Psalm cxxxvii.]

"Come, humble sinner, in whose breast,"

["By Rev. Edmund Jones, a popular Welsh minister of the last century."]

"Till I learned to love Thy name,
Lord, Thy grace denying,
I was lost in sin and shame,—
Dying, dying, dying.

"Henceforth shall creation ring,
With salvation's story,
'Till I rise with Thee to sing,
Glory, glory, glory."

Preceded by the note ["By Alice Cary, re-arranged for this collection."]

"Praise God from whom all blessings flow,"

etc., is the last verse of a hymn ["by Bishop Ken, of England, born 1667, died 1711, written for the Winchester school; this last stanza has probably been sung more frequently than any other four lines in the language, and is, by some of the best critics, pronounced the finest doxology in the English tongue."]

We have given a large space to this old subject of

PSALMS, AND HYMNS, AND SPIRITUAL SONGS, but music is the worship of man, the pastime of the birds, and the delight of the angels. It is soul-inspiring, it is health-giving. The man who can take a hymn-book and read it and sing it by the hour, has within him a well-spring of happiness worth all the gold in creation, and the practice will be a solace in sorrow, and a balm in troublous times, better than that which was Gilead's boast, and we can easily see how a Christian heart could while away many a happy hour with Deems and Cary's hymn-book, singing, and reciting, and meditating alternately, and all the while turning the eyes and the heart and the affections heavenward, lovingly, humbly trustingly.

